# Supplementary material for: Metabolic Alterations Caused by Simultaneous Loss of HK2 and PKM2 Leads to Photoreceptor Dysfunction and Degeneration
Source: Cells. 2023 Aug 10;12(16):2043. doi: 10.3390/cells12162043 (PMC10453858; doi:10.3390/cells12162043)

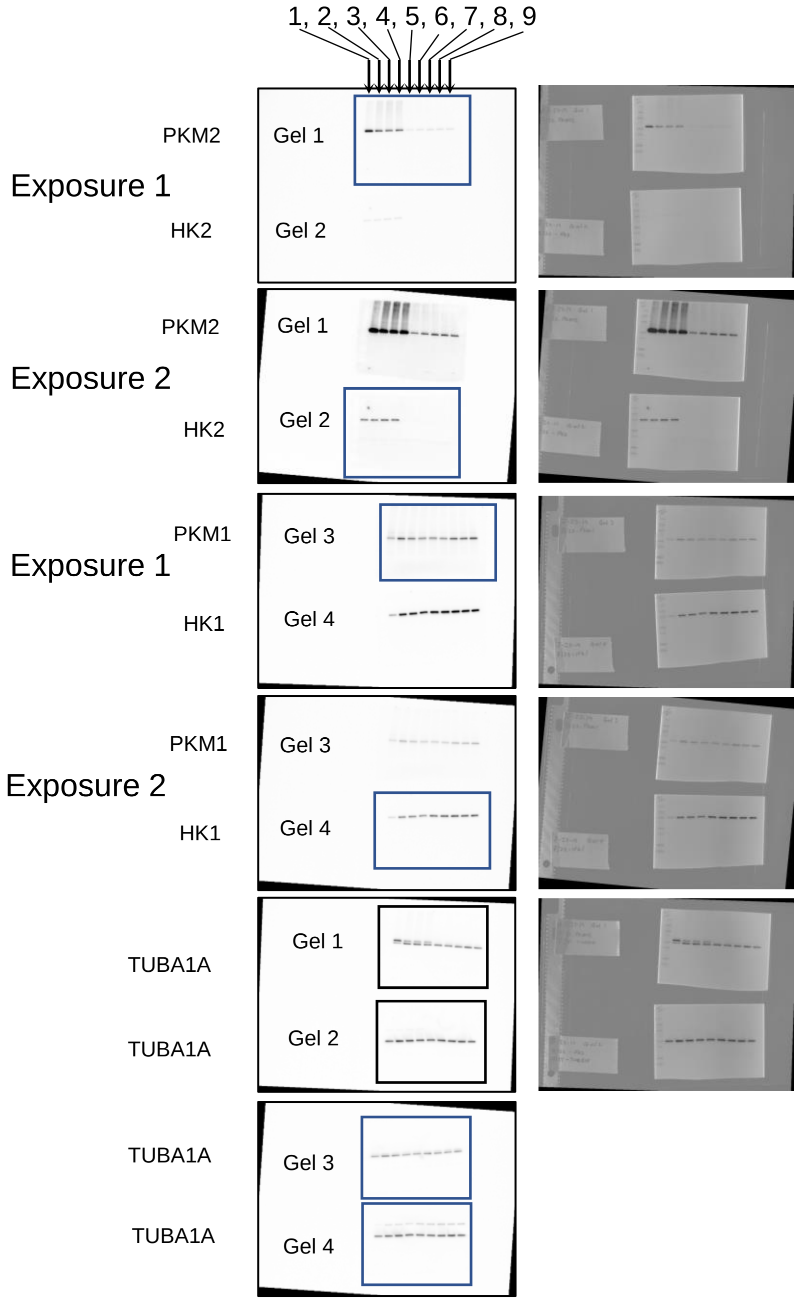

Supplemental Figure S1 – Figure 1A full Western images with molecular weight marker overlay.

1 – WT Sample #1 – excluded
2 – WT Sample #2
3 – WT Sample #3
4 – WT Sample #4
5 – dcKO Sample #1
6 – dcKO Sample #2
7 – dcKO Sample #3
8 – dcKO Sample #4
9 – dcKO Sample #5

Boxed region on each gel indicates exposure used for quantitation

MW Marker (Bio-rad Dual Color, Cat #1610374)
1 – 250kDA
2 – 150 kDa
3 – 100 kDa
4 – 75 kDa
5 – 50 kDa
6 – 37 kDa
7 – 25 kDa
8 – 20 kDa
9 – 15 kDa

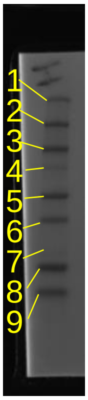

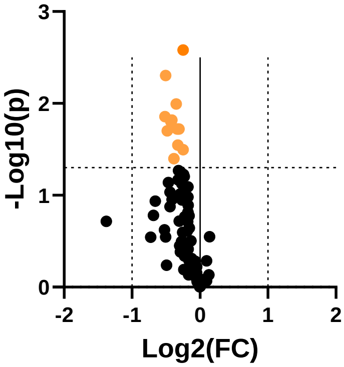

Gene	P-value	Fold Change
Aco1	0.04	0.77
Aldoa	0.03	0.84
Bpgm	0.00	0.70
Dlst	0.02	0.79
G6pdx	0.00	0.84
Gsk3b	0.03	0.80
Hk2	0.01	0.70
Ogdh	0.02	0.63
Pdha1	0.02	0.81
Pdk3	0.02	0.75
Pgm2	0.02	0.72
Tpi1	0.01	0.74

Supplemental Figure S2 – (A) Volcano plot showing significantly altered gene expression of genes related to central glucose metabolism as measured using qRT-PCR in 2-month old dcKO retinas. n = 4 per group. Vertical dashed lines indicate a fold change of ± 2- fold. Horizontal dashed line indicates a significance of $p \leq 0.05$. (B) Table showing significantly altered genes.

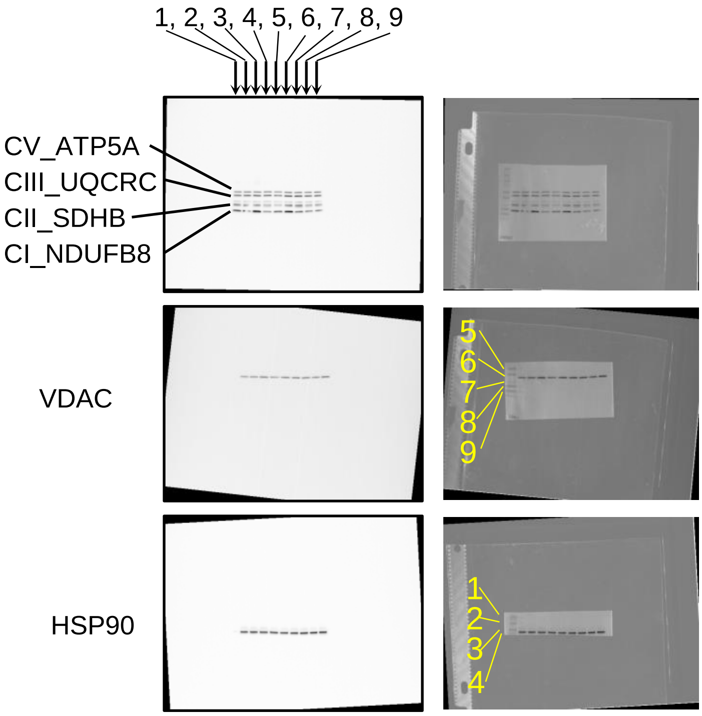

Supplemental Figure S3 – Figure 6C full Western images with molecular weight marker overlay.

1 – WT Sample #1
2 – WT Sample #2
3 – WT Sample #3
4 – WT Sample #4
5 – dcKO Sample #1
6 – dcKO Sample #2
7 – dcKO Sample #3
8 – dcKO Sample #4
9 – dcKO Sample #5

MW Marker (Bio-rad Dual Color, Cat #1610374)

1 – 250kDA
2 – 150 kDa
3 – 100 kDa
4 – 75 kDa
5 – 50 kDa
6 – 37 kDa
7 – 25 kDa
8 – 20 kDa
9 – 15 kDa

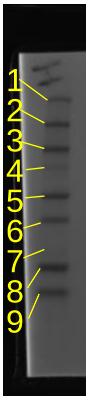

Supplement: Supplementary file 1 [file cells-12-02043-s001.zip › Supplemental Figures.pdf]
